# Intracellular Photophysics of an Osmium Complex bearing an Oligothiophene Extended Ligand

**DOI:** 10.1002/chem.202002667

**Published:** 2020-10-14

**Authors:** Kilian R. A. Schneider, Avinash Chettri, Houston D. Cole, Katharina Reglinski, Jannik Brückmann, John A. Roque, Anne Stumper, Djawed Nauroozi, Sylvia Schmid, Christoffer B. Lagerholm, Sven Rau, Peter Bäuerle, Christian Eggeling, Colin G. Cameron, Sherri A. McFarland, Benjamin Dietzek

**Affiliations:** ^1^ Department Functional Interfaces (K.R.A.S., A.C., B.D.) Department Biophysical Imaging (K.R., C.E.) Leibniz Institute of Photonic Technology (IPHT) e. V. Albert-Einstein-Straße 9 07745 Jena Germany; ^2^ Institute of Physical Chemistry and Abbe Center of Photonics Friedrich-Schiller-University Jena Helmholtzweg 4 07743 Jena Germany; ^3^ Department of Chemistry and Biochemistry The University of Texas at Arlington Arlington TX 76019-0065 USA; ^4^ Institute of Applied Optic and Biophysics Friedrich-Schiller University Jena Max-Wien-Platz 1 07743 Jena Germany; ^5^ University Hospital Jena Bachstraße 18 07743 Jena Germany; ^6^ Institute of Inorganic Chemistry I Ulm University Albert-Einstein-Allee 11 89081 Ulm Germany; ^7^ Department of Chemistry and Biochemistry The University of North Carolina at Greensboro Greensboro North Carolina 27402 USA; ^8^ Institute of Organic Chemistry II and Advanced Materials Ulm University Albert-Einstein-Allee 11 89081 Ulm Germany; ^9^ <MRC Human Immunology Unit & Wolfson Imaging Center Oxford Headley Way Oxford OX3 9DS UK

**Keywords:** in vitro spectroscopy, oligothiophene, osmium polypyridyl, transient absorption, ultrafast spectroscopy

## Abstract

This contribution describes the excited‐state properties of an Osmium‐complex when taken up into human cells. The complex **1** [Os(bpy)_2_(IP‐4T)](PF_6_)_2_ with bpy=2,2′‐bipyridine and IP‐4T=2‐{5′‐[3′,4′‐diethyl‐(2,2′‐bithien‐5‐yl)]‐3,4‐diethyl‐2,2′‐bithiophene}imidazo[4,5‐*f*][1,10]phenanthroline) can be discussed as a candidate for photodynamic therapy in the biological red/NIR window. The complex is taken up by MCF7 cells and localizes rather homogeneously within in the cytoplasm. To detail the sub‐ns photophysics of **1**, comparative transient absorption measurements were carried out in different solvents to derive a model of the photoinduced processes. Key to rationalize the excited‐state relaxation is a long‐lived ^3^ILCT state associated with the oligothiophene chain. This model was then tested with the complex internalized into MCF7 cells, since the intracellular environment has long been suspected to take big influence on the excited state properties. In our study of **1** in cells, we were able to show that, though the overall model remained the same, the excited‐state dynamics are affected strongly by the intracellular environment. Our study represents the first in depth correlation towards ex‐vivo and in vivo ultrafast spectroscopy for a possible photodrug.

## Graphical abstract

**Intracellular excited‐state properties** of a photodynamic active Osmium‐complex are investigated. The key to the excited‐state relaxation is the interplay of ^3^MLCT and ^3^ILCT states on the oligothiophene‐substituted ligand. We successfully tested the model derived from solution with the complex internalized in cells to bridge the gap between cuvette and in vivo measurements for the first time.

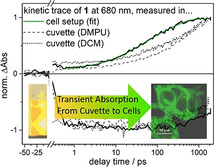

Transition‐metal complexes, and in particular Ru^II^ complexes,[^1^, ^2^, ^3^] are being investigated as photodrugs both in photodynamic therapy (PDT)[^4^, ^5^, ^6^, ^7^] and photochemotherapy (PCT).[^8^, ^9^, ^10^, ^11^, ^12^] Both approaches rely on the administration of an agent with relatively low cytotoxicity in the dark, which, becomes orders of magnitude more toxic upon irradiation with light. Only PDT has been approved clinically, and its underlying mechanism of action involves the generation of cytotoxic singlet oxygen (^1^O_2_) and other reactive oxygen species (ROS).[^13^, ^14^, ^15^] PCT has focused on avoiding this oxygen dependence mainly through photoinduced ligand loss and subsequent covalent modification of biomolecules as an alternate mechanism.[^16^, ^17^, ^18^, ^19^, ^20^, ^21^, ^22^, ^23^, ^24^, ^25^] While the Ru^II^ systems that have been investigated for PDT and PCT absorb visible light, many cannot be activated with wavelengths in the so‐called biological window (650–850 nm) that are desirable for deeper tissue penetration.

One strategy for shifting the optical window of metal complex phototherapy agents to longer wavelengths while building on successful molecular design concepts for Ru^II^ complexes is to utilize the Os^II^ analogues of the Ru^II^ complexes.[^26^, ^27^, ^28^, ^29^, ^30^] In fact, Os^II^ complexes are becoming widely appreciated as both therapeutic compounds[^31^, ^32^, ^33^, ^34^, ^35^, ^36^, ^37^, ^38^, ^39^, ^40^, ^41^] and cellular imaging agents.[^42^, ^43^, ^44^, ^45^, ^46^, ^47^] This approach has been explored by McFarland and co‐workers for PDT,^[48]^ but it is has not been applied to PCT given that the Os^II^ polypyridyl counterparts are photoinert.[^49^, ^50^] Both in vitro and in vivo studies of the Os^II^ photosensitizers **OsH2B, OsH2IP,** and **OsH2dppn**) (Scheme 1) published by McFarland and co‐workers showed panchromatic activation, low dark toxicity, and PDT activity.[^48^, ^51^]

**Scheme 1:**
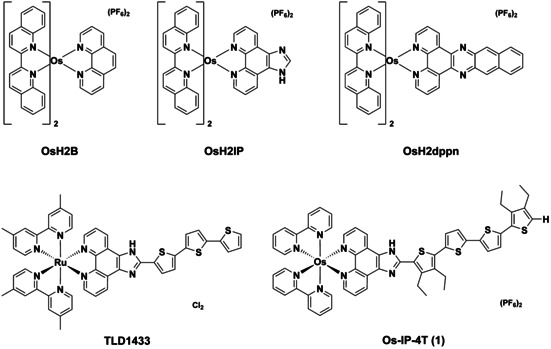
Molecular structures of osmium and ruthenium compounds previously investigated for PDT and Os‐IP‐4T (**1**) of this study. The compounds are racemic mixtures of the Δ/Λ isomers.

Building on this work, this study details the intracellular photophysics of the novel complex **Os‐IP‐4T** (**1**, [Os(bpy)_2_(IP‐4T)](PF_6_)_2_ with bpy=2,2′‐bipyridine and IP‐4T=2‐{5′‐[3′,4′‐diethyl‐(2,2′‐bithien‐5‐yl)]‐3,4‐diethyl‐2,2′‐bithiophene}imidazo[4,5‐*f*][1,10]phenanthroline), which was inspired by the Ru^II^ complex **TLD1433** ([Ru(4,4′‐dmb)_2_(IP‐3T)]Cl_2_ where 4,4′‐dmb=4,4′‐dimethyl‐2,2′‐bipyridine and IP‐3T=2‐(2′,2′′:5′′,2′′′′‐terthiophene)‐imidazo[4,5‐*f*][1,10]phenanthroline). **TLD1433** was developed by McFarland and co‐workers[^52^, ^53^, ^54^] and is currently in Phase II human clinical studies for treating bladder cancer with PDT.^[55]^ Our goal was to interrogate the impact of the biological target on the photophysics of the complex by studying the intracellular photophysics of **1** in MCF7 cells and comparing to the cell‐free environment. We carried out a detailed cell‐free photophysical study on the PF_6_^−^ salt of **1** in MeCN in order to be able to compare to other Os^II^ compounds in the literature that use this salt form and solvent and to determine how the aqueous intracellular photophysical properties compare to this standard cell‐free condition. The photophysical models proposed from the experimental data in this study assume predominantly one form of the potentially ionizable imidazo group of **1**.^[56]^

This comparative study is of particular relevance as the intracellular photophysics governs whether a compound will have photocytotoxic effects following light absorption. To truly understand the photoactivation mechanism, the light‐induced processes must not only be examined in solution but also within cancer cells. The results presented suggest a model of the excited‐state dynamics involving emissive triplet metal‐to‐ligand charge transfer (^3^MLCT) states, which enable tracking of the intracellular localization of the complexes, and dark triplet intraligand charge transfer (^3^ILCT) states that efficiently sensitize ^1^O_2_. The mechanistic work discussed herein highlights the importance of the π‐extended oligothienyl‐appended IP ligand in designing photoactive metal complexes with uniquely balanced ^3^MLCT and ^3^ILCT excited states for phototoxic effects.

The absorption spectra of **1** in the solvents dichloromethane (DCM) and acetonitrile (MeCN) (Figure 1 A) are dominated by overlapping ^1^ILCT and ^1^MLCT transitions between 390 nm and 525 nm as well as a less intense feature extending up to 700 nm, which originates from direct ^3^MLCT ← S_0_ transitions.[^57^, ^58^, ^59^] The aerated complex in MeCN is weakly emissive (*Φ*<0.1 %) with a maximum at around 735 nm. The emission excitation spectrum reflects the absorption spectrum with the exception of a minimum at 455 nm, which falls in the range of the IP‐4T ligand absorption with a band maximum at 405 nm extending to 500 nm (Figure 1 A). To further rationalize this feature, resonance Raman (rR) spectra were excited at 405 and 473 nm (Figure 1 B) and normalized to the MeCN band at 1374 cm^−1^. By comparison to the rR spectra of [Os(bpy)_3_]^2+^, **1** reveals specific bands for both bpy and IP‐4T. The intensity of the bpy‐related bands (e.g., at 1608 and 1556 cm^−1^) increased slightly upon excitation at 473 nm. However, the most prominent band is at 1452 cm^−1^, which is not present in the homoleptic complex. This band increased by 23 % with 473 nm excitation and was assigned to the symmetrical C=C stretching mode in quaterthiophene according to the literature.^[60]^ Together with the minimum in the emission excitation spectra at around 450 nm, this indicates a somewhat more prominent excitation of ILCT states, in which the excited‐state transition is localized on the IP‐4T ligand.

**Figure 1:**
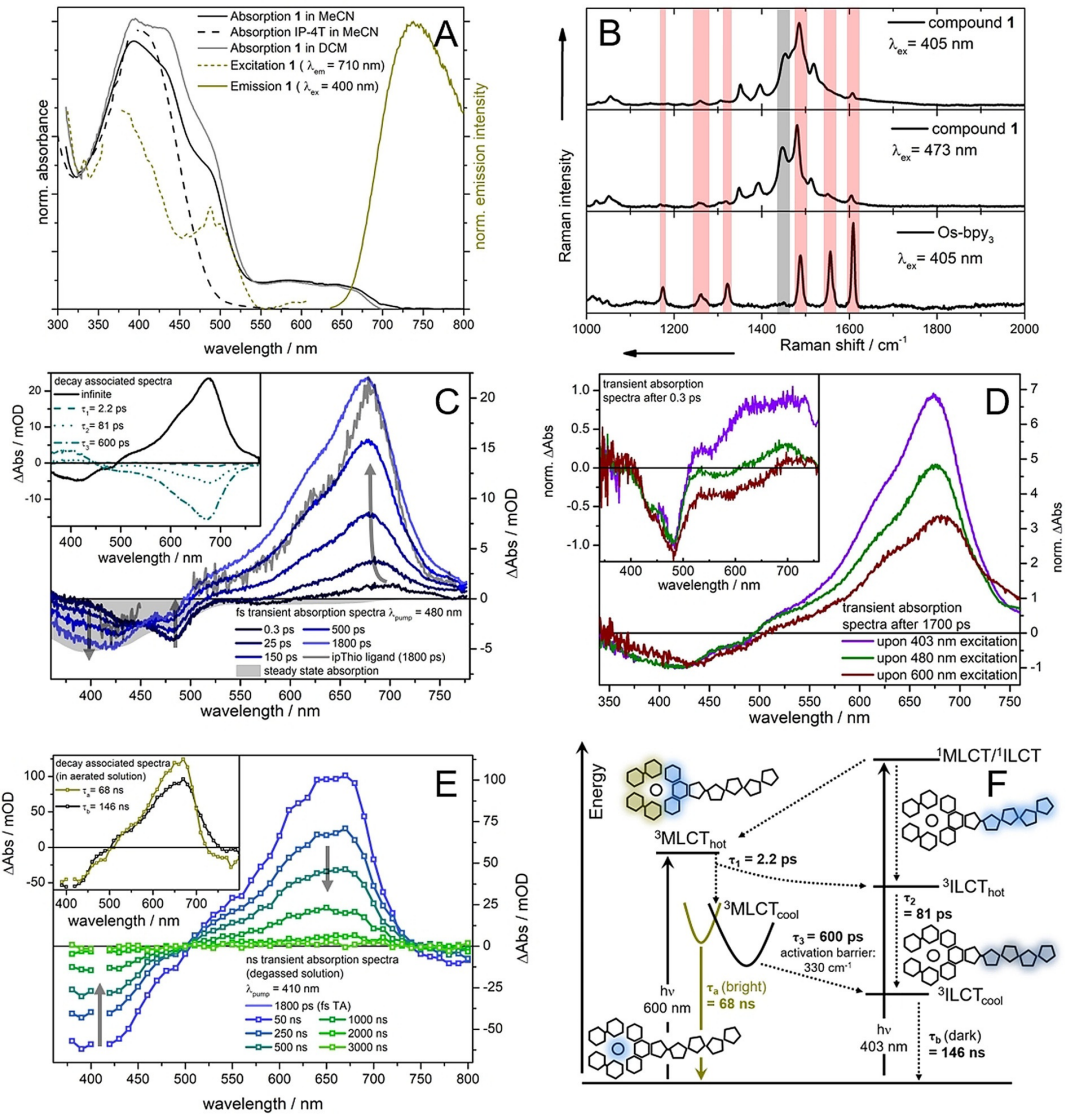
A) Steady state absorption spectra of complex **1** in DCM (grey) or ACN (black) as well as the steady state emission spectrum (*λ*_ex_=400 nm) in ACN (dark yellow) and excitation spectrum (*λ*_em_=710 nm) in ACN (dotted line, dark yellow). Normalized to the minimum at 325 nm (absorption and excitation) or maximum at 740 nm (emission). B) Resonance Raman (rR) spectra of **1** in ACN and [Os(bpy)_3_]^2+^ in water at 473 nm and 405 nm excitation. The red and grey lines indicates bpy and quaterthiophene‐related Raman bands, respectively. C) Femtosecond transient absorption spectra of **1** in ACN with *λ*_ex_=480 nm at different times, with filled and scaled steady state absorption spectrum as reference for GSB as well as the transient absorption spectrum of the free IP‐4T ligand at 1800 ps in grey. As inset the respective decay associated spectra of complex **1**. D) Transient absorption spectra of **1** in ACN at 1700 ps after excitation at 403 nm (violet), 480 nm (olive) and 600 nm (maroon), as inset the respective spectra at 0.3 ps delay time. Both normalized to the minimum of the GSB. E) The ns transient absorption spectra of **1** in degassed ACN solution (*λ*_ex_=410 nm) at different times. As inset the decay associated spectra of the ns transient absorption in aerated solution. F) Proposed model of the excited state dynamics of **1**. The bright deactivation pathway is depicted in yellow, while the dark is shown in black.

To investigate the most common mode of light‐driven cytotoxicity, the capacity of **1** to generate ^1^O_2_ was examined. Monitoring the ^1^O_2_ emission in the presence of **1** and referencing against [Ru(bpy)_3_](PF_6_)_2_ (in MeCN, *λ*_ex_=450 nm) resulted in a quantum yield for ^1^O_2_ production of 41 %, which is lower than that for comparable Ru^II^ complexes, but twice as high as certain other published [Os(bpy)_2_(LL)]^2+^ complexes.^[61]^ The singlet‐oxygen sensitization in organic solvent points to ILCT states being sufficiently long‐lived for photosensitization of ^1^O_2_. However, to understand the effect of the cellular environment and how this impacts the formation and lifetime of the triplet state responsible for ^1^O_2_ sensitization, we first required a complete picture of the excited‐state dynamics, including information on the ultrafast formation of the emissive excited state, in cell‐free solution. Thus, we begin with the investigation of the photoinduced dynamics in different solvents and then present the experiments in cells.

To investigate the photoinduced dynamics outside the Franck‐Condon region, femtosecond transient absorption (TA) spectra were recorded for **1** upon excitation at 480 nm (Figure 1 C, see Figure 1 F for the proposed model). Below 550 nm a negative signal centered at 480 nm is visible, which decays as a minimum at 420 nm develops. This negative differential absorption signal resembles the shape of the ground‐state absorption spectrum. Thus, at early delay times the ground‐state bleach (GSB) is superimposed by an excited‐state absorption (ESA) signal at about 400 nm, typically observed for of Os^II 3^MLCT states,^[31]^ which subsequently decays. We postulate that at early delay times, a triplet ^3^MLCT‐state is present, which upon blue excitation emerges from an initial ^1^MLCT/^1^ILCT manifold and decays with a characteristic time constant of *τ*_3_=600 ps as determined via a global fit of all kinetic traces. The ^3^MLCT‐decay does not cause decay of the GSB. Hence, the ^3^MLCT decay forms a secondary excited state, which is visible in the transient absorption spectra as the increasing ESA signal at 680 nm. The band at 680 nm, which emerges with *τ*_3_=600 ps, correlates to the long‐lived differential absorption feature of the free IP‐4T ligand (Figure 1 C) and agrees well with the TA signature of oligothiophenes.[^62^, ^63^] Thus, we assign the long‐lived species observed for **1** to an IP‐4T‐ligand centered ^3^ILCT state.^[64]^ However, even at early delay times, the transient absorption spectrum shows a characteristic feature at 690 nm pointing to the fact that almost immediately after photoexcitation, both ^3^MLCT and ^3^ILCT states are populated.

The kinetic analysis of the transient absorption data as reflected in the decay associated spectra (DAS) shows changes in the spectral region above 600 nm at early delay times (Figure 1 C). These changes can be related to the kinetic processes associated with the characteristic time constants *τ*_1_=2.2 ps and *τ*_2_=81 ps, i.e., on time scales where hardly any change is observed below 550 (Figure 1 C). *τ*_1_=2.2 ps leads to an increase of the 690 nm ESA band, likely due to a rapid partial population of the ^3^ILCT state from vibrationally hot ^3^MLCT states. This vibrationally hot ^3^MLCT state can also decay to the thermally relaxed ^3^MLCT_cool_ state through a much slower channel (*τ*_3_=600 ps), which subsequently populates the geometrically relaxed ^3^ILCT_cool_ state (vide supra). The process associated with *τ*_2_=81 ps leads to an increased ESA signal at 680 nm, which blue shifts (Figure S1 in the Supporting Information) and sharpens. A monoexponential fit of the position of the ESA maximum as a function of delay time yields a characteristic time constant of 81 ps, which is in good agreement with *τ*_2_ as determined from a global fit. Hence, *τ*_2_ was assigned to structural reorganization of the oligothiophene chain in the electronically excited state (^3^ILCT_hot_→^3^ILCT_cool_), which could involve planarization based on fact that oligothiophenes generally adopt the more rigid and coplanar quinoidal form in the excited state.^[65]^

The ratio of GSB (below 500 nm) and the ^3^ILCT‐associated ESA (between 600 and 700 nm) recorded at a delay of 0.3 ps changes as a function of excitation wavelength. Upon 600 nm excitation, where direct population of the ^1^ILCT state would not be expected, a transient absorption spectrum resembling the transient spectrum of [Os(bpy)_3_]^2+^ is observed.^[31]^ In contrast, excitation at 403 nm causes a strong ^3^ILCT‐associated ESA signal to become visible (Figure 1 D). At long delay times, i.e., 1700 ps, a pronounced ESA signal at 690 nm associated with the reorganized ^3^ILCT_cool_ state is observed irrespective of the excitation wavelength. This indicates that the population initially placed into the ^3^MLCT_cool_ channel is transferred to the ^3^ILCT_cool_ state on a longer time scale. The relative growth of the ^3^ILCT_cool_‐associated ESA band is larger with longer excitation wavelengths and this qualitative behavior does not depend on the polarity of the solvent (Table S1). Except for a prolongation of *τ*_1_=4.7 ps at 403 nm excitation, no significant changes of the characteristic time constants with excitation wavelength are observed.

To further investigate the photoinduced processes, time‐resolved data were recorded as a function of temperature as well as solvent polarity (dielectric constant, *ϵ*) and viscosity (*μ*). While these variations cause changes in the rates of the respective kinetic processes, the overall picture of the transient data remains unchanged (Table S3). Increasing the viscosity of the solvent on going from MeCN (*ϵ*=35.7; *μ*=0.39 cP) to *N*,*N′*‐dimethylpropyleneurea (DMPU) (*ϵ*=36.1; *μ*=3.41 cP) slows the overall excited‐state dynamics, whereby *τ*_2_, associated with the structural reorganization of the thienyl rings, increases from 81 to 132 ps. The conformational flexibility of the thienyl rings of the IP‐4T ligand at room temperature is hindered in the more viscous solvent, leading to a prolonged *τ*_2_ and generally slower excited state dynamics. The prolongation of *τ*_3_ in the more viscous DMPU suggests that the oligothiophene chain must adopt a certain geometry in order for energy transfer from the vibrationally relaxed ^3^MLCT_cool_ to the ^3^ILCT_cool_ state to occur. The energy barrier for this ^3^MLCT_cool_→^3^ILCT_cool_ transition was estimated from temperature‐dependent TA experiments in MeCN (see Figure S2). The prolonged *τ*_3_ that resulted from decreasing the temperature to 230 K yielded an energy barrier for the ^3^MLCT→^3^ILCT_cool_ transition of 4.0 kJ mol^−1^ (i.e., 330 cm^−1^).

This estimate for the barrier is significantly lower than the energy barrier that was estimated for interligand MLCT_bpy_‐to‐MLCT_bpy_ electron transfer in [Os(bpy)_3_]^2+^ (850 cm^−1^).^[66]^ Therefore, we assume that relaxation of the ^3^MLCT state via the ^3^ILCT_cool_ state is favored over population of the secondary bright MLCT state. Decreasing the solvent polarity from MeCN (*ϵ*=35.7; *μ*=0.39 cP) to DCM (*ϵ*=8.93; *μ*=0.42 cP), lengthened the lifetime of τ_1_ from 2.2 ps in MeCN to 6.9 ps in DCM (Table S3). This rate change was analyzed in the context of Marcus‐type electron transfer, whereby the ^3^ILCT_hot_ state is stabilized relative to the ^3^MLCT_hot_ state in MeCN. The driving force is larger and hence the reaction occurs with a faster rate and thus has a shorter characteristic time constant.

The excited‐state dynamics were also characterized by nanosecond TA spectroscopy (Figure 1 E). A global fit of the nanosecond TA data yielded two characteristic time constants, i.e., *τ*_a_=68 ns and *τ*_b_=146 ns. The emission lifetime determined by time‐correlated single‐photon counting (TCSPC) measurements (*τ*_em_=58 ns) agreed with the value for *τ*_a_ from the nanosecond TA data, and a comparison of the emission properties (Table S2) of **1** with those of [Os(bpy)_3_]^2+^ and the free ligand confirmed that emission from **1** was from the ^3^MLCT state. Therefore, the short component (*τ*_a_=68 ns) in the nanosecond TA experiments was assigned to the ^3^MLCT_cool_ state.

The ^3^MLCT‐based emission that decays on a 60 ns timescale in concert with the 600 ps depopulation of the ^3^MLCT_cool_ to the ^3^ILCT_cool_ state suggest two different and distinct decay pathways for the ^3^MLCT_cool_ state. We propose a model, where two conformers in the ^3^MLCT_cool_ manifold exist, one of which is able to populate the ^3^ILCT_cool_ state, while the other is decoupled from the thiophene chain and returns radiatively to the ground state. A similar conformer‐driven double potential was also seen for a pyrene‐substituted Ru complex.^[56]^ The ^3^ILCT_cool_ state on the other hand decays through a single pathway by intersystem crossing to the ground state with *τ*_b_=146 ns.

The full photophysical model is summarized in Figure 1 F. Photoexcitation populates a mixture of MLCT and ILCT states, and the MLCT/ ILCT ratio is determined by the excitation wavelength. Shorter excitation near 400 nm favors initial population of both ^1^MLCT and ^1^ILCT states while longer wavelength excitation near 600 nm initially populates the ^3^MLCT state exclusively. The vibrationally hot ^3^MLCT state, which is either excited directly upon 600 nm excitation or formed by very rapid inter system crossing upon blue excitation,[^66^, ^67^] partially decays into the ^3^ILCT state (*τ*_1_). Relaxation within the ^3^ILCT manifold involves structural reorganization of the oligothiophene chain. This process, which is associated with *τ*_2_, increases electronic delocalization within the oligothiophene and causes spectroscopic shifts of the characteristic ^3^ILCT_cool_ absorption band at around 680 nm. The fraction of molecules remaining within the thermally relaxed ^3^MLCT state decays to the ^3^ILCT_cool_ state on a sub‐ns timescale (*τ*_3_) or the decoupled conformer decays radiatively (*τ*_a_). The ^3^ILCT_cool_ on the other hand shows a dark ground‐state recovery (*τ*_b_).

To understand fully how **1** might act as a phototherapy agent, it is necessary to understand its photoinduced dynamics in the human cancer cells. While the localization of many photoactive transition metal complexes have been studied by luminescence microscopy in human cells,[^68^, ^69^, ^70^, ^71^] we are not aware of any detailed investigations of the photoinduced excited‐state dynamics of such photodrugs in these biological environments. To investigate the impact of the cellular environment on the photophysics, MCF7 cells were dosed with **1**, fixed and then spectroscopically interrogated. Confocal AiryScan fluorescence microscopy images after an 18 h incubation period revealed that the complex was taken up by cells and may accumulate preferentially in the cytosol and Golgi apparatus rather than the nucleus or lysosomes as it shows no enhanced co‐localization with the lysosomal marker LAMP1 (Figure 2). However, more in‐depth live‐cell investigations would be necessary to study possible long‐term lysosomal accumulation. This however is beyond the scope of this proof‐of‐concept study, in which we use fixed cells, where formalin fixation could disrupt organelle membranes and alter distribution of **1**.

**Figure 2:**
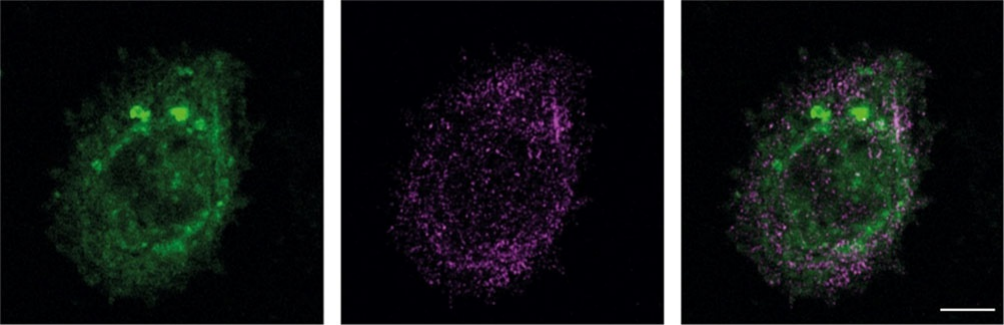
Cellular accumulation of **1**. Representative AiryScan images of MCF7 cells, incubated with **1** (25 μl mL^−1^) (green) for 18 h, and fixed and immunolabeled for the lysosomal marker protein LAMP1 (magenta): complex **1** (green, left), lysosomes (magenta, middle) and overlay (right). Scale bar 5 μm.

Fluorescence lifetime microscopy images (FLIM) on these samples showed an average emission lifetime of 23.5 ns irrespective of the region of the cytosol interrogated (Figure S3). This lifetime agreed with the emission lifetime of **1** in aqueous solution with 3 vol% DMSO (*τ*_em_=25 ns). This indicates that the properties of the long‐lived emissive ^3^MLCT state are not (strongly) affected by the cellular environment and fixation.

To study the ultrafast dynamics of **1** in MCF7 cells, a home‐built instrument was constructed to, for the first time, record time‐resolved differential absorption on fixed cells dosed with **1**. This equipment allows us to compare the photodriven response of **1** in cell‐free solution with that in cells. For the transient absorption experiments, intracellular **1** was excited at 403 nm and the photoinduced dynamics were studied at various probe wavelengths in the visible range (see caption of Figure 3 for details). Based on differential optical densities recorded at individual probe wavelengths, the shape of transient absorption spectra can be estimated (Figure 3 A). The estimated spectra agree well (within the range of the limited “spectral resolution”) with the transient absorption spectra recorded for **1** in cell‐free solution, indicating that the overall nature of the excited states is not impacted by the local environment provided by the fixed MCF7 cells in this particular example.

**Figure 3:**
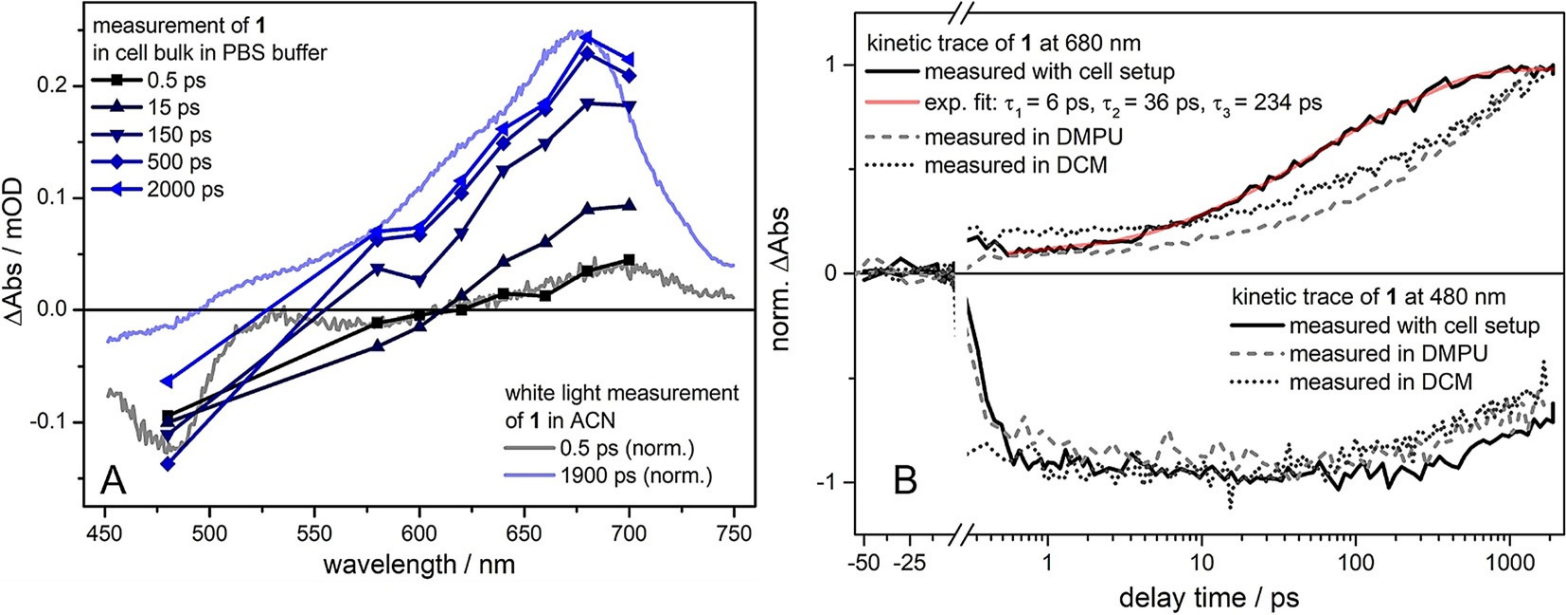
A) Femtosecond transient absorption spectra (*λ*_ex_=400 nm) of complex **1** measured with cell setup at different times (line with symbol), probed at 480, 580, 600, 620, 640, 660, 680, and 700 nm and probed white light, measured in MeCN (normalized, solid line). B) Kinetic trace of **1** at 480 nm and 680 nm measured with cell setup (black solid line), three‐exponential fit of 680 nm (red), as well as DMPU and DCM (grey, dashed, respectively dotted lines) of white light measurements.

The excited‐state relaxation kinetics were fit to a three‐exponential model. Figure 3 B shows the kinetic trace recorded at 680 nm, which is modeled by a sum of exponentials yielding the characteristic time constants *τ*_1_=6 ps, *τ*_2_=36 ps and *τ*_3_=243 ps. These characteristic time constants are in the same range as those observed for **1** in cell‐free solution, further corroborating the finding that internalization of the complex into fixed cells does not impact the ultrafast dynamics qualitatively.

Cellular values for *τ*_2_ and *τ*_3_ are about half of the respective time‐constants *τ*_2_=80 ps and *τ*_3_=600 ps obtained from measurements in solvents. The associated molecular processes were attributed to the structural reorganization of the oligothienyl group. The fact that the first‐order rates associated with the structural reorganization (*τ*_2_) as well as the energy transfer from the thermalized ^3^MLCT_cool_ to the ^3^ILCT_cool_ state of the reorganized oligothiophene chain (*τ*_3_) are increased by a factor of roughly two indicating rigidification of the oligothiophene chain. This could be associated with intermolecular interactions between the complex and the (macro)molecular constituents of the cells. While contributions from macromolecular cross‐linking and gelation of the cytosol associated with cell fixation cannot be excluded to contribute to the effect at this stage of the study, we assume that such nonspecific fixation effects would manifest themselves similar to an increase in solvent viscosity. Nonetheless, the intracellular kinetics are very different from those measured in the highly viscous solvent, which could point to cell‐specific interactions being responsible for the prolongation of *τ*_3_.

The intracellular ultrafast spectroscopy performed on fixed cells demonstrates proof‐of‐concept and is the necessary first step towards quantifying the ultrafast excited‐state dynamics in living cells. These first proof‐of‐concept studies showed an accelerated population of the ^3^ILCT_cool_ state. Such an effect may prove beneficial for photoinduced toxicity since the long‐lived ^3^ILCT_cool_ state facilitates ROS generation.^[72]^ The excited‐state dynamics observed for intracellular **1** on a sub‐ns time scale and its emission suggest that specific interactions between the complex and intracellular constituents do not alter the general excited‐state model for the complex, but that these interactions do have the capacity to influence the efficiency with which certain states are populated and in turn the ensuing biological properties. Thus, it is even more important to understand the photophysical dynamics inside living cells as the next step, and current efforts are underway to adapt the first‐of‐its‐kind transient absorption setup for cell bulk measurements for making these less static, more difficult measurements.

This study presents a complete picture of the ultrafast photophysics of complex **1**, a cell‐penetrating, red‐light absorbing Os^II^‐based photosensitizer. With in‐depth femto‐ and nanosecond TA measurements in a cell‐free environment, we were able to ascertain that the intracellular excited‐state dynamics are not significantly impacted by the biological milieu. In both cases, the photophysics are dominated by the interplay between MLCT and ILCT states.

Photoexcitation produces a mixture of ^1^MLCT/^1^ILCT or the triplet ^3^MLCT states that ultimately decay to a structurally reorganized longer‐lived ^3^ILCT_cool_ state within several hundred picoseconds. Some fraction of the excited population remains in a decoupled, bright ^3^MLCT_cool_ state that decays radiatively. We are able to correlate the established photophysical model in solution to that in a complex cellular environment, which is the relevant environment for understanding any light‐activated cytotoxicity.

The data shows that the interplay between ^3^ILCT states associated with the oligothiophene chain and the ^3^MLCT state determines the formation of long‐lived excited states that are most important for ROS production. Our study revealed that the emission lifetime of the complexes in fixed MCF7 cells is uniformly distributed and is in agreement with the emission lifetime of the complex recorded in aqueous solution as well as in organic solution.

These findings and the new experimental set‐up for measuring intracellular photophysics reported herein pave the way for future studies aimed at understanding how the cellular environment impacts the excited state dynamics of photosensitizers used for light‐based therapies. Our future studies involve a system upgrade that will utilize microscope incubators at the sample position in order to measure living cells, thus circumventing any possible alteration of natural cell environment due to fixation. The capacity to perform intracellular photophysical studies is a prerequisite to understanding the excited‐state dynamics and relating these photophysical processes to a compound's photo‐triggered biological activity, which will be evaluated in systematic studies to come.

## Experimental Section

Experimental details can be found in the Supporting Information.

## Conflict of interest

The authors declare no conflict of interest.

## Supporting information

As a service to our authors and readers, this journal provides supporting information supplied by the authors. Such materials are peer reviewed and may be re‐organized for online delivery, but are not copy‐edited or typeset. Technical support issues arising from supporting information (other than missing files) should be addressed to the authors.

Supplementary
