# Supplementary material for: Intracellular Photophysics of an Osmium Complex bearing an Oligothiophene Extended Ligand
Source: Chemistry. 2020 Oct 14;26(65):14844–51. doi: 10.1002/chem.202002667 (PMC7704931; doi:10.1002/chem.202002667)
Supplement: Supplementary file 1 — Supplementary [file CHEM-26-14844-s001.pdf]

Chemistry–A European Journal

Supporting Information

Intracellular Photophysics of an Osmium Complex bearing an Oligothiophene Extended Ligand

Kilian R. A. Schneider,[a, b] Avinash Chettri,[a, b] Houston D. Cole,[c] Katharina Reglinski,[a, d, e] Jannik Brückmann,[f] John A. Roque, III,[c, g] Anne Stumper,[f] Djawed Nauroozi,[f] Sylvia Schmid,[h] Christoffer B. Lagerholm,[i] Sven Rau,[f] Peter Bäuerle,[h] Christian Eggeling,[a, d, i] Colin G. Cameron,[c] Sherri A. McFarland,*[c] and Benjamin Dietzek*[a, b]

Table of Contents

Experimental Procedures

1. Synthesis and Characterization
2. Sample Preparation for Spectroscopic Experiments
3. Steady State Spectroscopy and Emission Lifetimes
4. Singlet Oxygen Generation
5. Femtosecond Transient Absorption Spectroscopy
6. Nanosecond Transient Absorption Spectroscopy
7. Temperature-dependent Transient Absorption Measurements
8. Resonance Raman Spectroscopy
9. Cell Cultivation and Sample Preparation
10. Fluorescence Lifetime Imaging Microscopy
11. Airyscan Microscopy and Co-Localization Experiments
12. Transient Absorption Measurements on Cell Bulk Samples

Results and Discussion

Experimental Procedures

1. Synthesis and Characterization

General remarks. Unless noted otherwise, all chemicals were commercially available and used without further purification. All solvents of technical grade were redistilled through a rotary evaporator prior to use. Solvents of higher quality grade were used without further purification. If not stated otherwise all reactions were carried out under normal laboratory conditions and without the use of absolute solvents. The following chemicals were prepared according to literature procedures: $[OsCl_2(bpy)_2]$,[1] 1,10-phenanthroline-5,6-dione,[2] and 3,3''',4,4'''-tetraethyl-[2,2':5',2'':5'',2'''-quaterthiophene.[3] Column chromatography was performed on Silica Gel 60 with acetonitrile or acetonitrile/water/KNO_3 mixtures.

NMR spectra were recorded on a Bruker AVANCE 400 spectrometer at 298 K. All spectra were referenced to the deuterated solvent as an internal standard. Chemical shifts are given by the residual solvent proton signal. Supramolecular investigations were performed by differing the concentration of the distinct sample. Coupling constants *J* are presented as absolute values in Hz, without considering the kind of the coupling. For the characterization of the NMR signals the following abbreviations are used: s = singlet, d = doublet, t = triplet, q = quartet, m = multiplet, dd = doublet of doublets, dt = doublet of triplets, td = triplet of doublets, ddd = doublet of doublet of doublets.

MALDI-MS values are given as *m/z*. Mass spectra were measured with MALDI-TOF equipment (Bruker Daltonics REFLEX III) and the added matrix was DCTB (trans-2-[3-(4-tert-Butylphenyl)-2-methyl-2-propenylidene]malononitrile).

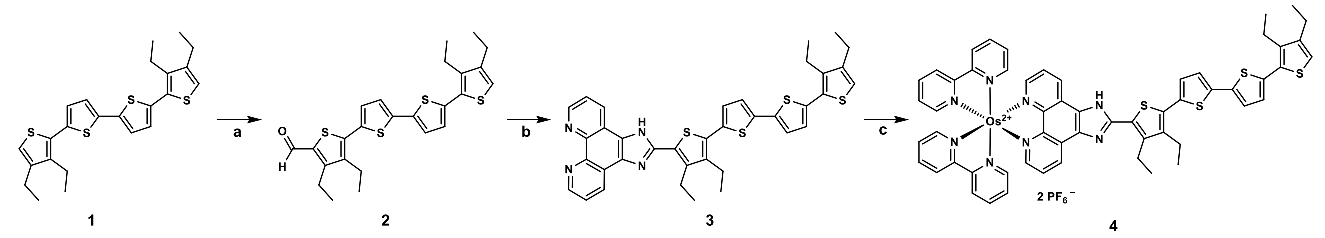

Scheme 1. Synthesis of 3,3''',4,4'''-tetraethyl[2,2':5',2'':5'',2'''-quaterthiophene]-5-carbaldehyde (**2**), 2-(3,3''',4,4'''-tetraethyl-[2,2':5',2'':5'',2'''-quaterthiophen]-5-yl)-1H-imidazo[4,5-f][1,10]phenanthroline (**3**) and corresponding Ru(II) and Os(II) complexes **4a**,**b**.

Synthesis of 3,3''',4,4'''-tetraethyl-[2,2':5',2'':5'',2'''-quaterthiophene]-5-carbaldehyde (4T-CHO) 2: The Vilsmeier-Haack reagent was prepared by dissolving 0.312 ml (1.8 eq., 4.0 mmol) DMF (abs.) and 0.366 ml (1.8 eq., 4.0 mmol) $POCl_3$ in 5 ml dichloroethane (DCE). After incubation, the reagent was added to 1.00 g (1 eq., 2.26 mmol) of quaterthiophene **1** in 10 ml DCE. The mixture was allowed to reflux for 2 hours. For work-up the reaction was quenched with saturated $NaHCO_3$ solution and diluted with DCM. The layers were separated and the organic layer was dried over sodium sulfate. After filtration, the organic layer was concentrated under reduced pressure. Purification was accomplished via column chromatography (silica gel) using DCM as eluent. The product was obtained as an orange solid (69%, 0.756 g, 1.61 mmol). ^1H-NMR (400 MHz, $CDCl_3$): **δ** = 10.03 (s, 1H), 7.21 (d, J = 3.8 Hz, 1H), 7.16 (dd, J = 6.1, 3.8 Hz, 2H), 7.05 (d, J = 3.8 Hz, 1H), 6.92 – 6.90 (m, 1H), 3.02 – 2.53 (m, 8H), 1.45 – 1.17 (m, 12H). ^{13}C-NMR (101 MHz, $CDCl_3$): δ = 181.88, 154.38, 144.99, 141.24, 141.03, 140.29, 138.95, 136.68, 135.89, 135.58, 133.82, 130.44, 128.15, 126.37, 124.53, 124.01, 118.95, 22.24, 20.85, 20.59, 20.44, 17.03, 15.11, 14.92, 13.76 ppm. HRMS (MALDI) *m/z* for $C_{25}H_{26}OS_4$ calc.: 470.08610. Found: 470.08604.

Synthesis of 2-(3,3''',4,4'''-tetraethyl-[2,2':5',2'':5'',2'''-quaterthien]-5-yl)-1H-imidazo[4,5-f][1,10]phenanthroline (IP-4T) 3: 1,10-Phenanthroline-5,6-dione (1.1 eq., 67.6 mg, 0.32 mmol), 3,3''',4,4'''-Tetraethyl-[2,2':5',2'':5'',2'''-quaterthiophene]-5-carbaldehyde (1 eq., 136.1 mg, 0.29 mmol) and ammonium acetate (10 eq., 223.3 mg, 2.90 mmol) were suspended in acetic acid (2 mL). The suspension was heated in a microwave at 300 W for 18.5 minutes. After cooling to room temperature, an aqueous solution of ammonia was added. The crude product was filtered off and washed with water, ethyl acetate and diethyl ether to give a brown solid of 2-(3,3''',4,4'''-tetraethyl-[2,2':5',2'':5'',2'''-quaterthiophen]-5-yl)-1H-imidazo[4,5-f][1,10]phenanthroline (56%, 103 mg, 0.16 mmol). ^1H NMR (400 MHz, DMSO-d_6) **δ** = 13.72 (s, 1H, H-Im), 9.09 – 9.03 (m, 2H, H-a/a'), 9.00 (dd, J = 8.2 Hz, J = 1.6 Hz, 1H, H-c/c'), 8.90 (dd, J = 8.1 Hz, J = 1.7 Hz, 1H, H-c'/c), 7.89 – 7.81 (m, J = 8.1 Hz, J = 4.0 Hz, 2H, H-b/b'), 7.43 (d, J = 3.8 Hz, 1H, H-T), 7.41 (d, J = 3.7 Hz, 1H, H-T), 7.32 (d, J = 3.9 Hz, 1H, H-T), 7.16 (d, J = 3.8 Hz, 1H, H-T), 3.21 (d, J = 7.3 Hz, 2H, H-CH2), 2.87 (d, J = 7.6 Hz, 2H, H-CH2), 2.72 (dt, J = 14.4, J = 7.3 Hz, 2H, H-CH2), 2.60 – 2.53 (m, 2H, H-CH2), 1.36 – 1.04 (m, 12H, H-CH3) ppm. ^{13}C NMR (126 MHz, 301.6 K, DMSO-d_6) **δ** = 148.34, 148.32, 146.11, 145.99, 145.20, 143.86, 143.80, 141.99, 140.46, 136.67, 136.42, 135.89, 135.69, 134.38, 130.88, 130.45, 130.24, 129.98, 127.74, 127.11, 126.34, 125.62, 125.56, 125.43, 124.04, 123.76, 123.71, 22.05, 21.40, 21.00, 20.75, 15.53, 15.48, 15.16, 14.21 ppm. HRMS (MALDI) *m/z* for $C_{37}H_{32}N_4S_4$ calc.: 661.15826. Found: 661.15746 ($[M+H]^+$).

Synthesis of Os-IP-4T $[PF_6]_2$ 4: 2-(3,3''',4,4'''-tetraethyl-[2,2':5',2'':5'',2'''-quaterthiophen]-5-yl)-1H-imidazo[4,5-f][1,10]phenanthroline (1 eq., 17 mg, 0.026 mmol) and $[Os(bpy)_2(Cl)_2]$ (1.1 eq., 16 mg, 0.028 mmol) were suspended in argon purged ethylene glycol (3 mL) and heated in a microwave (14 min, 600 W, 170 °C) to yield a greenish solution. After cooling, a saturated aqueous solution of ammonium hexafluorophosphate was added to precipitate the product. Purification via column chromatography (silica 60) was done starting with pure acetonitrile and ending with acetonitrile, water and potassium nitrate to yield $[Os(bpy)_2(IP\text{-}4T)][PF_6]_2$ (23 mg, 0.016 mmol, 62%). ^1H NMR (400 MHz, Acetonitrile-d_3) δ = 8.82 (d, J = 8.6 Hz, 2H, H-a), 8.50 (d, J = 8.3 Hz, 4H, H-3/3‘), 8.48 (d, J = 7.8 Hz, 4H, H-3/3‘), 7.96 – 7.87 (m, 4H, H-c,5/5‘), 7.83 – 7.74 (m, 4H, H-3/3‘/5/5‘), 7.67 (dd, J = 8.1 Hz, J = 5.5 Hz, 2H, H-b), 7.51 (dd, J = 6.0 Hz, J = 1.0 Hz, 2H, H-3/3‘), 7.39 – 7.33 (m, 2H, H-4/4‘), 7.20 – 7.15 (m, 2H, H-d/e), 7.15 – 7.10 (m, 2H, H-4/4‘), 7.05 – 6.99 (m, 2H, H-d/e), 3.32 – 3.17 (m, 2H, H-CH2), 2.91 – 2.80 (m, 2H, H-CH2), 2.80 – 2.71 (m, 2H, H-CH2), 2.64 – 2.55 (m, 2H, H-CH2), 1.38 – 1.11 (m, 12H, H-CH3). ^{13}C NMR (101 MHz, Acetonitrile-d_3) δ = 160.20, 159.96, 152.01, 151.98, 150.15, 149.43, 148.44, 147.98, 146.28, 142.62, 141.31, 138.07, 137.97, 137.63, 136.71, 135.13, 132.95, 130.91, 129.00, 127.92, 127.27, 126.93, 125.73, 125.42, 125.37, 125.01, 120.30. HR-MS (MALDI) *m/z* for $C_{57}H_{48}N_8OsS_4^+$ calc.: 1163.24158. Found: 1163.24265 ($[M\text{-}2PF_6\text{-}H]^+$).

2. Sample Preparation for Spectroscopic Experiments

Complex **1** was investigated spectroscopically by steady-state absorption, emission and femtosecond time-resolved transient absorption spectroscopy. The respective experiments were performed at room temperature in air-equilibrated solutions of acetonitrile (ACN), dichloromethane (DCM) or *N,N′*-dimethylpropyleneurea (DMPU). The solvents were obtained in spectroscopic-grade from Sigma Aldrich and used without further purification. All measurements were executed in quartz glass cuvettes with a path length of 1 mm at a concentration of approx. 0.05 mmol/L, yielding an optical density of 0.2 at 400 nm for transient absorption measurements. Emission measurements were performed in a 1 cm cuvette with an optical density of 0.1 at 400 nm. To investigate the effect of deprotonation, trimethylamine (TEA) was added to ACN. The bare IP-4T ligand was investigated in DCM due to its limited solubility in ACN.

3. Steady State Spectroscopy and Emission Lifetimes

A Jasco V-670 spectrophotometer was used for measuring the steady-state absorption spectra before and after each fs-transient absorption measurement to ensure sample integrity. Steady-state emission experiments were performed using a Jasco FP-6200 spectrofluorometer (λ_{ex} = 400 nm), while the emission quantum yield was determined with FLS980 spectrometer (Edinburgh Instrument) and $[Ru(bpy)_3]^2(Cl)_2$ as reference. The emission lifetimes were investigated using time-correlated single photon counting (TCSPC, Becker & Hickl GmbH) with laser excitation at 400 nm.[4]

4. Singlet Oxygen Generation

Singlet oxygen generation was measured by exploiting the phosphorescence of $^1O_2$ at 1275 nm. This was achieved by measuring the phosphorescence of a solution of **1** (in ACN, optical density = 0.05 at λ_{ex} = 450 nm) in the FLS980 spectrometer. The integrated area of the emission was compared with that of the reference solution of $[Ru(bpy)_3](Cl)_2$ (in ACN, optical density = 0.05, λ_{ex} = 450 nm, $\Phi(^1O_2)$ = 0.82).

5. Femtosecond Transient Absorption Spectroscopy

The femtosecond (fs) transient absorption experiments, at least three independent measurements for every set of parameters, were performed using a custom-build setup which is described in detail elsewhere.[5] The measurements were performed upon excitation at 403, 480 and 600 nm. The excited state dynamics were probed with a white-light supercontinuum generated by focusing a minor part of the amplifier output into a CaF_2 plate. The data was chirp-corrected and subsequently analyzed using a sum of exponential functions for a global fit. The temporal resolution of the experiment is limited to 300 fs due to strong contributions from coherent artifact signals to the data,[6,7] which impair the reliable analysis of the pump-probe data by means of multi-exponential fitting at shorter delay times. To study the solvent-dependence and viscosity-dependence of the light-induced dynamics, different solvents were used, which vary in polarity and dynamic viscosity: ACN (ε=35.7, μ=0.39 cP), DCM (ε=8.93, μ=0.42 cP), DMPU (ε=36.1, μ=3.41 cP).

6. Nanosecond Transient Absorption Spectroscopy

The nanosecond (ns) transient absorption and emission experiments were executed using a setup (Pascher Instruments AB) described previously.[8] In short, samples in a 1 cm quartz glass cuvette were excited at 410 nm and probed between 380 and

800 nm in 10 nm steps. The data was analyzed using a global fit of all probe wavelengths. Upon recording 720 nm without probe light, the emission lifetime of an aqueous solution of **1** could be measured using the ns transient system. For this, a sample with 3% DMSO and an optical density of 0.2 at 410 nm was prepared using a DMSO stock solution due to limited solubility of **1** in water.

7. Temperature-dependent Transient Absorption Measurements

For temperature-dependent measurements a cryostat (OptistatDN, Oxford Instruments) with liquid nitrogen cooling system was fit into the fs-transient absorption setup, replacing the regular sample holder. A special quartz glass cuvette with a path length of 1 cm was used in the cryostat, thus a lower concertation was applied to yield an optical density of 0.2 at the excitation wavelengths. The temperature was controlled using a temperature probe inside the sample.[9]

8. Resonance Raman Spectroscopy

Resonance Raman experiments were performed through excitation by either an 473 nm blue diode pumped solid state laser (HB-Laser, Germany) or a TopMode-405-HP diode laser (Toptica, Germany), and detected by an IsoPlane 160 spectrometer (Princeton Instruments, USA) with an entrance slit width of 0.05 mm, focal length 750 mm, and grating 2400 grooves/mm. The Raman signals were recorded using a thermoelectric cooling PIXIS eXcelon camera (Princeton Instruments, USA). Complex **1** was measured in ACN, while $[Os(bpy)_3](Cl)_2$ was measured in water due to solubility. 1 mm cuvettes were used and a transmission geometry was employed. The Raman spectra were baseline corrected, and normalized with respect to an acetonitrile solvent band, *i.e.*, to the signal at 1374 cm^{-1}. The acetonitrile spectrum was then subtracted from all resonance Raman spectra.

9. Cell Cultivation and Sample Preparation

For cell experiments MCF7 cells were incubated in RPMI 1640 medium (with L-glutamine, 10% foetal bovine serum and 2% streptomycin-penicilin). Splitting the cells was done when approximately 90% confluency was achieved with 0.25% trypsin/EDTA for 5 min at 37°C. For sample preparation, the cells were seeded into ibidi µ-dish^{35mm,high} glass bottom and incubated as above for 24 hours. As soon as a uniform growth of the glass bottom was observed, the cells were fixed with 5% formalin solution and then stained with **1**. The stock solution of **1** was prepared in DMSO due to the limited solubility of PF_6^- salt of **1** in water and the comparably low cytotoxicity of DMSO. The cells were stained for two hours in the dark with a 5% v/v DMSO in PBS buffer staining solution with a 10 µM concentration of **1**. Alternatively, the living cells in the µ-dish were incubated with **1** (0,5% v/v DMSO, final concentration of **1** was 1 µM) for 18 hours, washed twice with PBS and fixed afterwards. Knowing that the intracellular accumulation can be affected by the incubation time, we strictly applied the staining solution for 18 hours. For each experiment, at least three independently produced samples were used to ensure biological reproducibility.

10. Fluorescence Lifetime Imaging Microscopy

Time-Correlated Single Photon Counting (TCSPC) measurements were performed on a Leica SP8 STED 3X (Leica, Mannheim, Germany) system additionally equipped with Single Molecule Detection (SMD) software (SymPhoTime version 5.3.2.2) and hardware (PicoHarp 300; PHR 800, Picoquant, Berlin, Germany). Excitation in this system was with a pulsed (20 MHz) tuneable white light laser (WLL; Super-K; NKT Photonics, Birkerød, Denmark) while emission detection was with an internal hybrid single molecule detector (HyD SMD Leica, Mannheim, Germany). Fluorescence excitation was performed at a wavelength of 488 nm with an emission detection band of 648-750 nm and using a 100X 1.4 NA oil-immersion objective (HC PL APO 100x/1.40 Oil STED WHITE, Leica, Mannheim, Germany) with a zoom factor of 3.26X, a scan speed of 400 Hz, and a pinhole setting of 3 Airy units. TCSPC data were analyzed using SymPhoTime software (v2.1, Picoquant, Berlin, Germany). Cell preparation was as outlined in j.

11. Airyscan Microscopy and Co-Localization Experiments

MCF7 cells, incubated with **1** for 18 h, were fixed with 3% formaldehyde in PBS, permeabilized for 5 min with 0.1% Triton X-100, and blocked with 2% BSA + 5% FCS in PBS for 1 h at room temperature. Samples were incubated with primary antibodies against the lysosomal marker protein Lamp1 (polyclonal antibody rabbit, Fisher Scientific) diluted 1:200 in 1% BSA in PBS for 1 h at room temperature. After several washing steps, the cells were incubated for 30 min with secondary antibodies conjugated to Alexa Fluor 568 (Fisher Scientific) diluted 1:250 in 1% BSA in PBS. The samples were washed again and then mounted in Mowiol.

The samples were imaged with a Zeiss 980 LSM with AiryScan 2 (Zeiss, Oberkochen, Germany) to investigate the subcellular localization of complex **1**. Here the AiryScan was mainly used to increase the contrast in the images. Complex **1** was exited with the

488 nm laser and its emission detected between 658 and 720 nm, the Alexa Fluor 568 was exited with the 561nm laser and its emission was detected from 574 to 627 nm. A 63x/1.40 oil objective was used.

12. Transient Absorption Measurements on Cell Bulk Samples

A home-built transient absorption setup was used to perform measurements in a cell bulk. Pump and sample beam were generated from an amplified Ti:Sapphire oscillator (Libra, Coherent Inc.), producing a 80 fs 950 µJ pulse centered at 800 nm with a repetition rate of 1 kHz. By splitting the pules with a 50:50 beamsplitter we can simultaneously produce the pump pulse by means of second harmonic generation in a 100 µm thick BBO crystal and also pump a non-collinear optical-parametric amplifiers (TOPASwhite, LightConversion Ltd.) to generate the probe pulse in a wavelength region between 500 and 700 nm. With a chopper every second pump pulse was blocked. The probe pulse was sent over a delay line to introduce a time delay of these pulses with respect to the pump pulse and a 30:70 beam splitter was used to obtain reference and sample pulses. The probe sample beam was focused with a 400 mm quartz lens from below into a horizontal sample holder by means of a periscope. To excite with magic angle geometry, the polarization of the pump pulse was adjusted accordingly and then focused with a 750 mm quartz lens and an angle of 20° through the same periscope on the sample holder. The light scattered by the cells is focused into the detector by means of a large diameter lens (f = 100 mm), whereby scattered pump light is separated by means of a long pass filter (LP > 450 nm). Both reference and sample probe beams were recorded with photodiodes and electronically read out by the detections system provided by Pascher Instruments AB. In each overgrown petri dish transient absorption data were recorded in five different spatial positions.

Results and Discussion

As discussed in the paper, there is a pump-wavelength dependency of the excited-state relaxation, as seen in the transient absorption data upon excitation at 403, 480 and 600 nm. In Table S1 the ratio of ESA maximum at 690 nm versus the magnitude of GSB minimum below 500 nm at these three excitation wavelengths in ACN and DCM is summarized, as well as the percentage growth of this ratio. A high ratio is an indication for ILCT state population, which has a strong ESA feature at around 690 nm compared to the much weaker MLCT contribution there. Therefore the ILCT state is initially, at 0.3 ps, stronger populated at 403 nm (ratio 0.6) then at 600 nm (ratio 0.2). However, if the percentage growth of this band is compared for different excitation wavelengths, it is noticeable that the growth after excitation at 600 nm is stronger than in the case of 403 nm excitation. This indicates that the population initially placed into the MLCT channel is transferred to the ILCT state on a longer time scale. Thus, the percentage growth of the band is stronger upon 600 nm excitation compared to excitation at 480 and 403 nm. This behavior does not depend on the polarity of the solvent and can also be observed in DCM as well.

Table S1. Overview of ratio of ESA maximum at 690 nm versus magnitude of GSB minimum below 500 nm at different excitation wavelengths in ACN and DCM.

	λ_{ex}	Ratio at 0.3 ps	Ratio at 1800 ps	Growth in %
ACN	403 nm	0.6	5.5	820
	480 nm	0.4	4.7	1075
	600 nm	0.2	3.1	1450
DCM	403 nm	0.7	3.9	460
	480 nm	0.5	4.1	720
	600 nm	0.2	4.2	2000

To rationalize the emission properties of **1**, emission spectra, lifetimes and quantum yields were determined in different solvents and compared to the homoleptic complex $[Os(bpy)_3]$ $(PF_6)_2$ (in ACN) and the free IP-4T ligand (in DCM). The results are shown in Table S2. It can be seen that the emission properties of **1** differ strongly from the bare ligand, however they resemble very much the properties of the homoleptic complex. This gives a strong argument for a MLCT-based emission compared to alternative, which would be the radiative decay of a ILCT-state on the IP-4T ligand.

Table S2. Overview of emission wavelengths, lifetimes and quantum yields of **1**, the IP-4T ligand and $[Os(bpy)_3]^{2+}$ in different solvents.

λ_{ex} = 410 nm (solvent)	λ_{em} / nm	τ_{em} / ns	Φ_{em} / %
**1** (ACN)	735	58	< 1
$[Os(bpy)_3]^{2+}$ (ACN)[10]	740	54	1.19

IP-4T ligand (DCM)	495	0.4	≈ 24

To verify the model proposed in the paper, the ultrafast light-induced processes were investigated, whereby specific parameters were changed, *i.e.* polarity and viscosity and temperature. Though these alterations in the experimental parameters impose significant differences on the rates of the respective processes, the overall transient picture of the transiently populated states remains the same (see Table S3).

Table S3: Overview of the lifetimes obtained by a global fit of the transient absorption data as measured with different parameters.

**Chosen solvent and parameter**	**τ_1 / ps**	**τ_2 / ps**	**τ_3 / ps**
ACN (ε = 35.7; μ = 0.39 cps)	2.2	81	600
DCM (ε = 8.93; μ = 0.42 cps)	6.9	69	600
DMPU (ε = 36.1; μ = 3.41 cps)	13	132	1100
ACN 233K	ND	23	980

In order to investigate the transition from the hot ^{3}MLCT to the ^{3}ILCT state with τ_1, polarity dependent measurements were performed because the ^{3}ILCT state has a higher dipole moment then the ^{3}MLCT state and can therefore be better (de-)stabilized by the solvent. Measurements were performed at 480 nm excitation wavelength in ACN and DCM (see Table 3). Both solvents have similar viscosities but ACN (ε = 35.7; μ = 0.39 cps) is more polar than DCM (ε = 8.93; μ = 0.42 cps). The overall picture of the excited state dynamics is similar for the two solvents. However, the first lifetime τ_1 is significantly prolonged in DCM from 2.2 to 6.9 ps, while τ_2 and τ_3 do not show a significant change (see table S1). The fact, that the polarity dependence is only visible in τ_1 indicates that the hot ^{3}MLCT state is depopulated to the ^{3}ILCT. The prolongation of τ_1 is in agreement with electron transfer in the Marcus normal region, since the ILCT state will be energetically stabilized by the more polar solvent ACN, thereby increasing the gap between MLCT and ILCT and thus, by increasing the driving force, shortening τ_1 in ACN compared to DCM.[11]
In the model the process associated with τ_2 is the structural reorganization of the thiophene chain in the ^{3}ILCT state to yield the structural reorganized state we call ^{3}ILCT$_{cool}$. Since a flattening will increase the oscillator strength of transitions, an increase of the maximum of the ESA signal is to be expected, which can be seen in the DAS. However, due to energetically stabilization a blue shift of the ESA maximum can also be proposed. Figure S1 shows exactly this for a measurement in ACN at 480 nm excitation wavelengths.

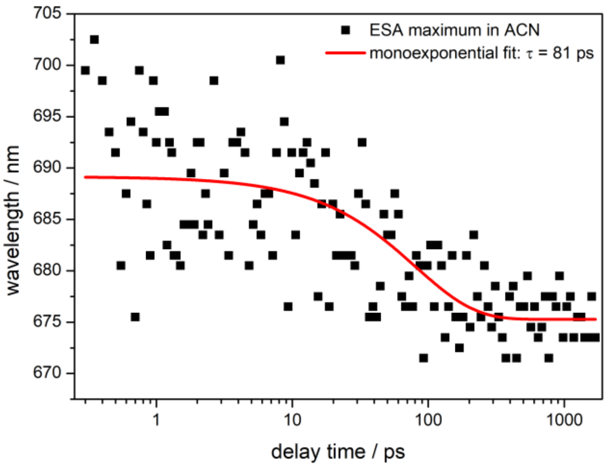

Figure S1: ESA maximum versus the respective delay time of complex **1** (in ACN, λ_{ex} = 480 nm). The blue-shift was fitted monoexponentially with the mean value of τ_2 = 81 ps, obtained *via* global fit.

The influence of viscosity was investigated by measurements in the highly viscous solvent DMPU at 480 nm excitation wavelength, whereby a prolongation of all three time constants was observed. Since the process to which τ_2 is assigned is a flattening which requires molecular motion, an extension of this time constant by a more viscous solvent is only natural. The extension of τ_3 can be explained by the fact that the transition to the reorganized ^{3}ILCT' state from the cool ^{3}MLCT requires a certain geometric ordering of the chain, which is less likely in the higher viscosity solvent and needs to be overcome by thermal activation.

The implication of the thermal activation of the process assigned to τ_3 led to the possibility of determining the activation energy of this transition by means of temperature-dependent measurements. Therefore measurements were performed in a cryostat in a temperature window of 233 to 303 K in ACN upon excitation at 480 nm. While the overall spectral features are preserved, τ_3 significantly increases upon lowering the temperature, resulting in τ_3 = 980 ps for the lowest measured temperature of 233 K. By means of Arrhenius plots and linear fit, the energy barrier of the transition from $^3MLCT_{cool}$ to $^3ILCT'$ can be estimated to be 4.0 kJ/mol (330 cm^{-1}, see Figure S2).

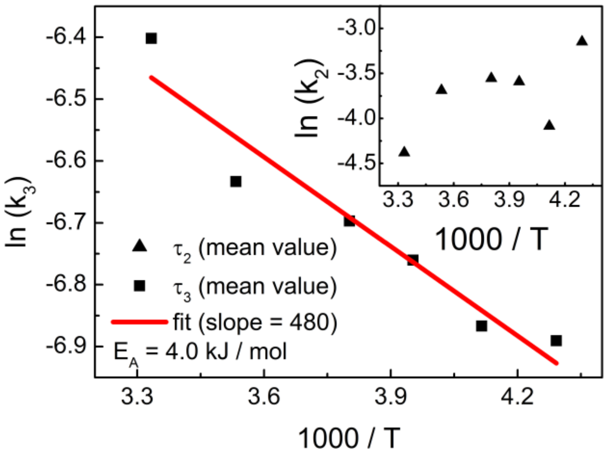

Figure S2: Arrhenius-plot for τ_2 (triangles) and τ_3 (squares) of **1** in the temperature interval from 303 K to 233 K (in ACN, λ_{ex} = 480 nm). Fitting of the τ_3 lifetimes (red line) yielded an activation energy of 4.0 kJ/mol (330 cm^{-1}).

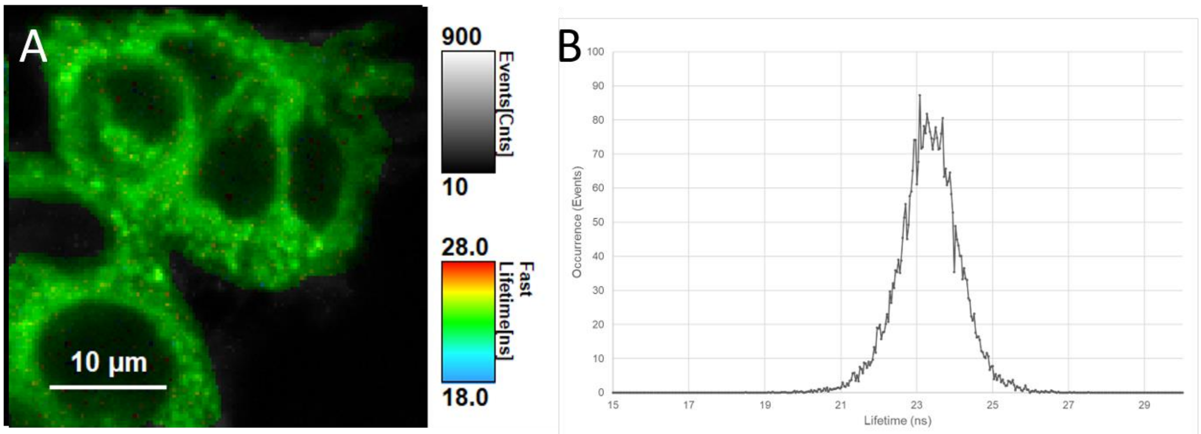

Figure S3: A) Representative fluorescence lifetime microscopy (FLIM) images (λ_{ex}= 488 nm) of MCF7 cells incubated with **1** (25µl/ml) for 18 h and then fixed, with color scale representing lifetime values as labeled. B) Histogram of all lifetime values of the pixels inside the cell with a mean intracellular emission lifetime of 23.5 ns.

References

[1] E. M. Kober, J. V Caspar, B. P. Sullivan, T. J. Meyer, *Inorg. Chem.* **1988**, *27*, 4587–4598.

[2] M. Yamada, Y. Tanaka, Y. Yoshimoto, S. Kuroda, I. Shimao, *Bull. Chem. Soc. Jpn.* **2006**, *65*, 1006–1011.

[3] S. Schmid, E. Marion Schneider, E. Brier, P. Bäuerle, *J. Mater. Chem. B* **2014**, *2*, 7861–7865.

[4] S. H. Habenicht, M. Siegmann, S. Kupfer, J. Kübel, D. Weiß, D. Cherek, U. Möller, B. Dietzek, S. Gräfe, R. Beckert, *Methods Appl. Fluoresc.* **2015**, *3*, 025005.

[5] R. Siebert, D. Akimov, M. Schmitt, A. Winter, U. S. Schubert, B. Dietzek, J. Popp, *ChemPhysChem* **2009**, *10*, 910–919.

[6] A. L. Dobryakov, S. A. Kovalenko, N. P. Ernsting, *J. Chem. Phys.* **2005**, *123*, 0445021–0445028.

[7] B. Dietzek, T. Pascher, V. Sundström, A. Yartsev, *Laser Phys. Lett.* **2007**, *4*, 38–43.

[8] M. Stephenson, C. Reichardt, M. Pinto, M. Wa, T. Sainuddin, G. Shi, H. Yin, S. Monro, E. Sampson, B. Dietzek, et al., *J. Phys. Chem. A* **2014**, *118*, 10507–10521.

[9] Y. Luo, J. H. Tran, M. Wächtler, M. Schulz, K. Barthelmes, A. Winter, S. Rau, U. S. Schubert, B. Dietzek, *Chem. Commun.* **2019**, *55*, 2273–2276.

[10] D. Liu, Y. Zhao, Z. Wang, K. Xu, J. Zhao, *Dalt. Trans.* **2018**, *47*, 8619–8628.

[11] R. A. Marcus, *J. Chem. Phys.* **1956**, *24*, 966–978.

Author Contributions

Kilian R. A. Schneider: lead experimental investigation, data analysis, lead writing of original draft
Avinash Chettri: supporting experimental spectroscopic investigation
Houston Cole: supporting writing and data analysis
Katharina Reglinski: supporting investigation (performed emission microscopy experiments) and data analysis, supporting writing
Jannik Brückmann: lead synthesis of the complex
John Roque III: supporting writing, supporting data analysis
Anne Stumper: supporting synthesis of the complex
Djawed Nauroozi: supporting synthesis of the complex and data analysis
Sylvia Schmid: lead synthesis of the ligand
Christoffer B. Lagerholm: supporting investigation, supporting writing
Sven Rau: developing concept of the study, planning the synthesis, data analysis, supporting writing
Peter Bäuerle: planning the synthesis, data analysis, supporting writing
Christian Eggeling: planning of emission microscopy experiments, data analysis and supporting writing
Colin G. Cameron: supporting data analysis, supporting writing
Sherri A. McFarland: data analysis, supporting concept of the study, writing of the manuscript
Benjamin Dietzek: funding acquisition, project administration, developing concept of the study, data analysis, writing
